# Clinical Applications and Efficacy of Cyanoacrylates in Oral Surgery and Periodontology: A Scoping Review

**DOI:** 10.3390/medicina61071286

**Published:** 2025-07-17

**Authors:** Davide Gerardi, Pierangelo Burdo, Ilser Turkyilmaz, Francesca Diomede, Gustavo Duarte Mendes, Maurizio Piattelli, Giuseppe Varvara

**Affiliations:** 1 Department of Innovative Technologies in Medicine & Dentistry, University “G. d’Annunzio” of Chieti-Pescara, 66100 Chieti, Italy; davide.gerardi@graduate.univaq.it (D.G.); pierangeloburdo@libero.it (P.B.); francesca.diomede@unich.it (F.D.); mpiattell@unich.it (M.P.); 2 Department of Life, Health and Environmental Sciences, University of L’Aquila, 67100 L’Aquila, Italy; 3 Department of Comprehensive Care, School of Dental Medicine, Case Western Reserve University, Cleveland, OH 44106-4905, USA; ilserturkyilmaz@yahoo.com; 4 Faculdade de Odontologia, Universidade Metropolitana de Santos, Santos 11000-000, Brazil; gustavo.mendes@unimes.br

**Keywords:** cyanoacrylates, tissue adhesive, bioadhesive, oral surgery, periodontology

## Abstract

*Background and Objectives*: Cyanoacrylate (CA) tissue adhesives have gained increasing attention as alternatives to sutures in oral surgery and periodontology. The objective of this scoping review is to assess their clinical applications and effectiveness in wound closure and postoperative management. *Materials and Methods*: The review was conducted following the JBI methodology and PRISMA-ScR guidelines. A comprehensive search was performed in PubMed, Scopus, and Web of Science to identify randomized controlled trials published between 2015 and 2025 evaluating the use of CAs in oral surgery and periodontal procedures. *Results*: A total of 19 studies were included. Cyanoacrylate adhesives demonstrated comparable or superior outcomes to other wound healing strategies in terms of operative time, postoperative pain reduction, and early wound healing. Their use was particularly beneficial in free gingival grafts and palatal donor site management. However, the findings across studies were not always consistent, and some trials did not report statistically significant differences. The use of long-chain CA formulations is associated with minimal toxicological risk, though these adhesives demonstrate intrinsic hemostatic and antimicrobial effects. *Conclusions*: Cyanoacrylate tissue adhesives represent a valid alternative to sutures in several dental surgical contexts, especially in procedures involving mucogingival grafts. Further high-quality clinical studies are needed to clarify their long-term outcomes and broaden their indications in dentistry.

## 1. Introduction

Oral mucosal wound healing involves a series of sequential biological processes that facilitate the closure of ruptures in this tissue [1]. Wound healing is conventionally divided into four phases: coagulation and hemostasis; inflammation; proliferation; and remodeling with scar tissue formation [2]. The inflammatory phase begins immediately following injury, characterized by homeostasis and inflammatory responses, and lasts approximately 4 to 6 days. The proliferation phase involves epithelialization, angiogenesis, granulation tissue formation, and collagen deposition, occurring from day 4 to day 14, with epithelial cell migration starting within 24 h. The maturation and remodeling phase starts around day 8 and can extend for up to a year, during which tissue organization and scar formation occur [3]. Wound management can be broadly categorized into three main approaches: primary closure with suture material; healing by secondary intention, whereby the wound heals naturally without surgical intervention; and healing by tertiary intention, which involves surgical closure of the wound following a period of secondary healing [4].

There are many approaches used for wound closure, such as sutures and bioadhesives, which may also be applied in combination with other wound closure methods, e.g., to prevent anastomotic leak [5].

Sutures are indispensable for wound closure, but they come with several limitations. These include secondary trauma to tissue caused by needle insertion, risk of inflammation, infection, or delayed healing, and risk of needle puncture accidents [6].

Other wound healing strategies include the use of platelet-rich fibrin (PRF) and collagen sponges, both of which support tissue regeneration and hemostasis. Likewise, tissue adhesives (e.g., cyanoacrylate glue) have been employed to achieve wound closure and bleeding control [7].

The term bioadhesive refers to tissue glues, films, and sealants that can be classified as either biodegradable or biostable [5]. Bioadhesives adhere to biological tissues when applied to their surfaces, effectively preventing separation by redistributing mechanical loads between tissues. Tissue adhesives can be divided into two main categories: natural polymer-based adhesives, such as fibrin, albumin, and gelatin; and synthetic adhesives, including those based on CA, poly(ethylene glycol) (PEG), catechol, and methacrylic anhydride [8].

Among bioadhesives, CAs represent a group of adhesives that have been widely used for closing skin wounds and in numerous surgical procedures involving skin, mucous membranes, and different tissues, such as oral tissues [9]. Cyanoacrylates are synthetic chemical adhesives that exhibit strong bonding capabilities and undergo rapid exothermic polymerization upon contact with basic substances (such as water, blood, or tissue moisture), forming a durable film within approximately 60 s [8]. The properties of CAs are primarily determined by the nature of their alkyl side chains. Based on the R group, CAs are classified into five main types: methyl (2-MCA), ethyl (2-ECA), n-butyl (nBCA), isobutyl (ICA), and 2-octyl (2-OCA) cyanoacrylates. These compounds are further categorized into short-chain and long-chain variants. Short-chain CAs degrade rapidly and are associated with higher cytotoxicity, limiting their medical use. In contrast, long-chain CAs exhibit slower degradation and significantly lower toxicity, making them more suitable for biomedical applications [8]. The CA monomer is synthesized through the condensation of formaldehyde and cyanoacetate molecules, yielding a polymer known as polycyanoacrylate. As this reaction is reversible, degradation occurs, producing formaldehyde monomers and cyanoacetate once more. It is suggested that this degradation pathway may be responsible for the toxic effects observed in human tissues [10].

Cyanoacrylates are characterized by immediate hemostatic and embolic effects, a long half-life, and rapid adhesion to both hard and soft tissues in moist environments [11]. Their ease of application contributes to reduced operation time, and they do not need to be removed during postoperative follow-up. Additionally, they are biodegradable and biocompatible, and also have antimicrobial properties in the oral cavity due to their bacteriostatic nature [12].

Cyanoacrylates are employed in various medical procedures, including skin closure, fixation of hernia meshes, and endoscopic obliteration of esophageal or gastric varices, and as embolic materials for arterial occlusion [13].

In dentistry, CAs have been employed in various surgical procedures, including the fixation of mucogingival grafts or membranes in guided tissue regeneration [14]. Cyanoacrylate adhesives have been successfully introduced in intraoral wound closure as a way to prevent the formation of ischemic areas resulting from sutures [15]. They also promote palatal healing after free gingival harvesting and may also serve as hemostatic agents in high-risk extraction cases, in particular those involving third molar removal [14].

We hypothesize that CA adhesives provide superior or comparable outcomes to other wound healing strategies, in particular sutures, in terms of postoperative pain, healing time, and patient comfort. The objective of this scoping review is to assess the clinical applications and the efficacy of CAs in oral surgery and periodontology.

## 2. Materials and Methods

This scoping review follows the JBI methodology for scoping reviews [16]. This project is registered as https://doi.org/10.17605/OSF.IO/EH6S4 (accessed on 31 May 2025) on OSF.

### 2.1. Research Questions

In order to fulfill the objectives of this scoping review, the following research questions are posed:

1. “What are the main clinical applications of CA tissue adhesives in the field of oral surgery and periodontology?” This question aims to explore the specific surgical and periodontal procedures in which CAs have been applied.
2. “How do CAs compare to conventional sutures in terms of clinical effectiveness and postoperative outcomes?” This question is intended to assess whether the use of CAs results in statistically or clinically significant differences regarding healing time, pain levels, patient comfort, and overall wound management.
3. “What are the advantages and limitations of CAs?” This question aims to identify common evidence reported in the literature regarding the strengths and limitations of CAs.
4. “Are there any reported adverse effects or concerns regarding the toxicity or biocompatibility of CA adhesives used intraorally?” This question focuses on evaluating the evidence related to the potential cytotoxicity, allergic reactions, or other complications associated with their use.
5. “In which contexts do CAs appear to be more effective or preferable to traditional methods of wound closure?” This question examines clinical contexts where CAs may be considered superior or equivalent to sutures.

### 2.2. Search Strategy

This scoping review searched three different databases, PubMed, Scopus, and Web of Science, to identify the relevant literature on the topic published between 2015 and 2025. A single comprehensive search strategy was developed to cover both oral surgery and periodontology. The review was conducted in accordance with the PRISMA for Scoping Review (PRISMA-ScR) guidelines [17].

### 2.3. Search Terms

An electronic search was conducted using the following search terms: (cyanoacrylates) AND ((dentistry) OR (oral surgery) OR (periodontology)).

The PCC (Population, Concept, Context) method was used to develop this scoping review, defining the following parameters:

P: population of living humans;

C: clinical applications and efficacy of cyanoacrylates;

C: oral surgery and periodontology.

### 2.4. Inclusion Criteria

Eligible studies were randomized controlled trials published in English in the last 10 years exploring the application and efficacy of CA tissue adhesive in oral surgery and periodontology performed in vivo on living humans.

### 2.5. Exclusion Criteria

The following study types were excluded: abstracts, chapters of books, non-peer-reviewed articles, conference proceedings, proceeding papers, clinical trials, comparative studies, cohort studies, case–control studies, case series, case reports, comprehensive reviews, narrative reviews, systematic reviews, meta-analyses, and studies not in English. We excluded non-English studies due to limited translation resources, although this may have introduced a language bias.

### 2.6. Study Selection

Two independent authors (D.G. and P.B.) carried out the primary literature search and then conducted a subsequent evaluation of the selected articles, excluding studies that did not meet the predefined eligibility and inclusion criteria. The full texts of the eligible studies were then reviewed to determine their suitability for final inclusion. Any disagreements between the reviewers were resolved through discussion and re-evaluation. The studies meeting all criteria were finally included in the qualitative synthesis. To assess inter-rater reliability during the study selection process, Cohen’s Kappa coefficient (κ) was calculated.

### 2.7. Critical Appraisal

Although quality assessment is not mandatory in scoping reviews, a simplified critical appraisal of the included randomized controlled trials using the JBI Checklist for RCTs was performed [18]. Each study was independently reviewed by two authors (D.G. and P.B.), with disagreements resolved by a third reviewer (G.V.). The checklist includes 13 criteria designed to highlight potential bias. No studies were excluded based on quality assessment outcomes.

## 3. Results

**Figure 1:**
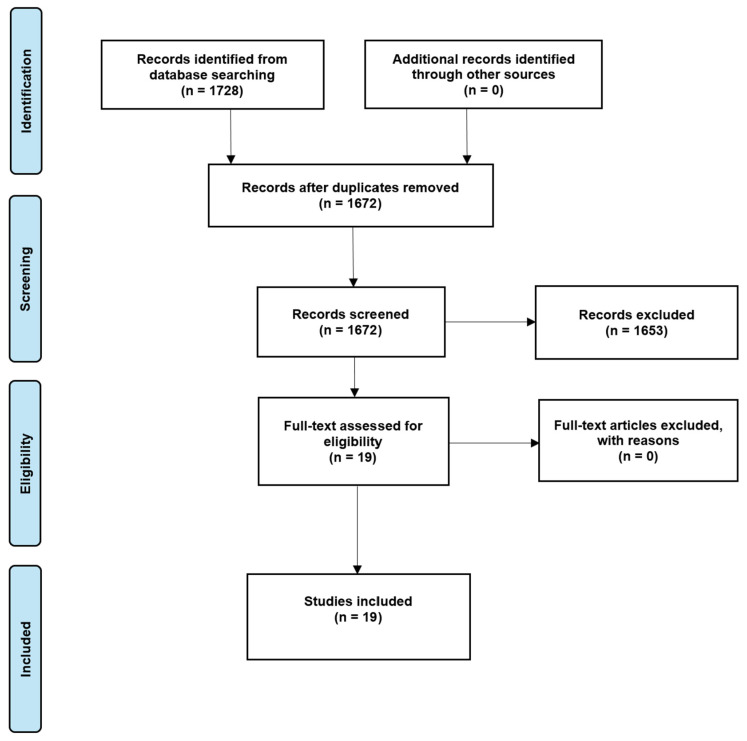
PRISMA flowchart illustrating the literature search and selection process used to identify studies on the clinical application and efficacy of CAs in oral surgery and periodontology.

The PRISMA diagram for the scoping review (Figure 1) illustrates the selection process used to identify studies exploring the clinical application and efficacy of CAs in oral surgery and periodontology. The initial search yielded 1728 potentially relevant articles. After screening the titles and abstracts, 1672 studies were reviewed. Nineteen full texts were assessed for eligibility and subsequently included in the scoping review. The inter-rater agreement was evaluated using Cohen’s Kappa coefficient, indicating almost perfect agreement between reviewers both in the preliminary screening phase (κ = 0.94) and in the full-text eligibility assessment (κ = 0.85). Finally, six studies were included after full-text evaluation. Table 1 presents the clinical applications of CAs in oral surgery and periodontology and provides a brief summary of each study included in the manuscript.

### 3.1. Stabilization and Fixation of Free Gingival Graft (FGG)

In the study by Alhourani et al., the aim was to compare the combination of two different CAs (N-BCA+OCA) and surgical sutures in the stabilization and fixation of free gingival grafts (FGGs). In total, 12 patients with gingival recession and the absence of keratinized gingiva were enrolled, making a total of 24 cases. They underwent FGG surgery at two parallel sites at the same time. One graft was sutured and the other was stabilized with CAs. The sites where the grafts were fixed with tissue adhesives exhibited less shrinkage compared to the other group after 3 months (*p* < 0.05) and a better healing index after 2 months. Additionally, the results showed that the postoperative pain ended in 3 days, while, in the sites fixed with sutures, it persisted for up to 4 days. Cyanoacrylates were demonstrated to be an effective alternative material to sutures in stabilizing the free gingival graft at the recipient site [19].

### 3.2. Wound Management at Palatal Donor Sites

In the study by Basma et al., four different wound dressings were compared after free epithelialized mucosal graft (FEG) harvesting in a randomized clinical trial involving 72 patients. The patients were split into a control group, which received a collagen plug combined with sutures (CPS), and three test groups: one received a collagen plug combined with CA (CPC), another was treated with platelet-rich fibrin (PRF) along with sutures, and, in the last group, only a palatal stent (PS) was used. All interventions significantly reduced pain perception compared to the application of a hemostatic collagen sponge alone at the palatal donor site after FEG (*p* < 0.0001), and no statistically significant differences among the test groups were found. The palatal stent (PS) group exhibited the lowest overall pain scores throughout the 14-day observation period [20]. Tavelli et al. evaluated the effects of various hemostatic treatments on patient-reported discomfort following palatal gingival graft harvesting. Fifty patients requiring mucogingival surgery were randomly allocated into five groups: (1) a control group treated with sutures alone; (2) a CA group; (3) a group receiving a periodontal dressing; (4) a group treated with a hemostatic gelatin sponge; and (5) a group treated with both a gelatin sponge and CA. Over a 14-day period, all experimental groups reported significantly lower pain levels (VAS) compared to the control group (*p*-value < 0.01 for time–group interaction). The combination of the gelatin sponge and CA caused minimal pain (VAS ≤ 0.5) throughout the observation period. At day 10, the control group had the lowest healing scores (VAS 6.8), whereas the test groups had scores that ranged from 8.2 to 9.0 (*p* = 0.0001). The application of a dual-layer hemostatic approach using both a gelatin sponge and CA was the most effective in minimizing postoperative pain and discomfort [21]. Another study by Tavelli et al. aimed to compare the effectiveness of a hemostatic collagen sponge alone versus a collagen sponge sealed with CA in reducing postoperative pain at palatal donor sites following epithelialized gingival graft (EGG) harvesting. A total of 44 patients undergoing EGG procedures were divided into two groups: the control group received only a collagen sponge on the palatal wound, whereas the test group received an additional CA layer. After a 14-day postoperative period, statistically significant differences in pain perception were observed between the groups (*p* < 0.01), with the test group consistently reporting lower pain levels. Analgesic consumption was also significantly reduced in the test group (*p* < 0.01), supporting the efficacy of CA application in minimizing postoperative discomfort [22]. In the study by Ozcan et al., the aim was to compare the effect of three different interventions on palatal wound healing. A total of 125 patients undergoing free gingival graft (FGG) harvesting were assigned to three groups: one which received PRF with butyl-CA (BC) adhesive (PRF group; n = 42), one which received the BC adhesive alone (BC group; n = 42), and one which received sterile wet gauze compression (WG group; n = 41). Statistically significant differences were found for all parameters (bleeding, pain, epithelialization, feeding habits, and sensation scores) in favor of the PRF group (*p* = 0.0001) [23]. The study by Stavropoulou et al. aimed to compare patient-reported outcomes, early wound healing, and postoperative complications at palatal donor sites following subepithelial connective tissue graft (CTG) harvesting when either CA tissue adhesive or polytetrafluoroethylene (PTFE) sutures were used for wound closure. The 36 patients were randomized into two groups: the suture group, treated with continuous interlocking 6-0 PTFE sutures, and the CA group, where a high-viscosity blend of n-butyl and 2-octyl CA was applied until hemostasis was achieved. No significant differences were observed between the groups regarding patient-reported discomfort during the first postoperative week (median VAS: 1.49 vs. 1.86; *p* = 0.56), patient self-reported pain on the first day and in the first week after surgery, analgesic consumption, or the modified early wound healing index (MEHI). However, the application time was significantly shorter for CA compared to sutures (2.16 vs. 7.31 *min*; *p* < 0.0001), indicating that cyanoacrylate may offer a time-efficient alternative for palatal wound closure without compromising clinical outcomes [24]. Yılmaz et al. assessed the impact of CA application on graft dimensions, clinical healing parameters, and patient-based outcomes at both the recipient and donor sites during free gingival graft (FGG) procedures. Participants undergoing FGG surgery were randomly assigned to a test or a control group. In the test group, graft stabilization was performed using CA, which was also applied to the donor site. In the control group, stabilization was achieved with 6-0 polyvinylidene fluoride sutures, and no material was applied to the donor site. No significant differences were found between the groups for any outcome, except for the horizontal dimension loss of the graft, which was significantly greater in the CA group at six months (*p* > 0.05 and *p* < 0.05, respectively). The findings suggest that CA is a safe alternative for use in FGG procedures, although it does not demonstrate superior outcomes compared to conventional suturing with polyvinylidene fluoride [25]. Karimi et al. conducted a randomized controlled clinical trial to evaluate the effects of CA adhesive on palatal wound healing following free gingival graft (FGG) harvesting. Fifteen patients requiring bilateral FGG were treated with sutures on one side (Vicryl 4-0) and CA (a blend of n-butyl and 2-octyl) on the contralateral side, and wound closure was found to be significantly faster with CA (2.12 ± 1.23 *min*) compared to sutures (8.42 ± 2.24 *min*; *p* < 0.05). Furthermore, at one week, the CA group had significantly lower pain scores (−1.54 units; 17%; *p* = 0.04), reduced discomfort (−1.18 units; 15.8%; *p* < 0.05), and lower analgesic intake (−2.12 tablets; 21%; *p* < 0.002), and experienced significantly better healing (+0.9 units; 22%; *p* < 0.05). Dimensional changes at one week were significantly greater in the suture group (+0.81 units; 28.9%; *p* < 0.01). These findings indicate that CA offers advantages in terms of operative time, early pain control, healing, and patient comfort following FGG harvesting [26].

### 3.3. Wound Closure After Mandibular Third Molar Surgery

Santmartí-Oliver et al. compared CA and 4-0 silk sutures for flap closure in mandibular third molar (M3M) surgery in a split-mouth randomized clinical trial. The surgery was performed bilaterally on 17 patients with a total of 34 M3Ms. No statistically significant differences were found in postoperative outcome measures (pain, swelling, trismus, and healing) or patient-reported outcome measures (PROMs) between the two groups (*p* < 0.05) [27]. Oladega et al. conducted a randomized clinical trial to compare postoperative outcomes and wound healing following surgical closure with either CA tissue adhesive or silk sutures in patients undergoing removal of mesio-angularly impacted mandibular third molars. The participants were randomly assigned to two groups: the control group received silk sutures, while the study group was treated with CA adhesive. A total of 60 subjects in each group completed the study. After a 7-day postoperative period, no significant differences were found between the two groups in terms of pain (*p* = 0.95), swelling (*p* = 0.66), trismus, wound dehiscence (*p* = 0.51), or infection (only two subjects (1.6%)). However, on postoperative day 1, the CA group exhibited significantly less bleeding compared to the suture group (*p* = 0.02) [28].

### 3.4. Wound Closure After Periodontal Flap Surgery

The objective of the study by Gautam et al. was to compare clinical outcomes following periodontal flap surgery using either 3-0 braided silk sutures or isoamyl 2-CA for wound closure. Twenty surgical sites from ten patients with moderate-to-severe periodontitis were randomly assigned to two groups after completion of phase I therapy: a test group that received isoamyl 2-CA and a control group that received 3-0 silk sutures. Postoperative healing was evaluated on days 3, 5, 7, and 14, with assessment of wound healing, pain on a verbal rating scale (VRS), and the number of analgesic tablets consumed. While no statistically significant differences were found between the groups in terms of VRS scores (*p* > 0.05) or wound healing (*p* > 0.05) at any time point, the test group required fewer analgesics postoperatively (*p* = 0.004 (day 3); *p* = 0.006 (day 5)). These findings suggest that isoamyl 2-CA may serve as a viable alternative to silk sutures, offering reduced postoperative pain and discomfort [29]. Gupta et al. conducted a randomized split-mouth clinical trial to evaluate the efficacy of two periodontal dressing materials (Coe-Pak™ and Xoin CA) on Porphyromonas gingivalis (*P. gingivalis*) levels and patient satisfaction following periodontal flap surgery. Eleven patients with generalized chronic periodontitis were enrolled, and three different quadrants were treated per patient: one with sutures alone, one with sutures followed by Coe-Pak™, and one with Xoin CA adhesive alone. All treatment modalities resulted in a significant reduction in P. gingivalis colony-forming units (CFUs) by day 7 (*p* = 0.001), with the greatest reduction observed in the CA group (mean difference = 1254.2). Pain scores were significantly lower in the CA group compared to the others (*p* = 0.001). The results indicate that Xoin CA is a biocompatible, effective alternative to conventional periodontal dressings, providing superior antimicrobial activity, reduced postoperative pain, greater patient acceptance, and efficient hemostasis in the context of periodontal flap surgery [30]. Chandra et al. aimed to evaluate the efficacy of N-butyl CA compared to 3-0 braided silk sutures in flap closure following periodontal surgery in 40 patients with moderate-to-severe generalized periodontitis. Patients were assigned to two groups: a test group treated with N-butyl CA and a control group treated with silk sutures. The CA group showed better early wound healing, with a significant difference observed on day 3. However, no statistically significant differences in wound healing were found between the groups on days 14 and 21. In terms of plaque accumulation and gingival inflammation, the CA group showed significantly better outcomes on days 3 and 7 (*p* < 0.05), but the differences between the groups were no longer significant by days 14 and 21 (*p* > 0.05). Postoperative pain was also significantly reduced in the CA group during the first 7 days (*p* < 0.05), and there was no subsequent significant difference in VAS scores between groups. N-butyl CA appears to be a reliable alternative to conventional sutures, providing better early wound healing, less early plaque accumulation, reduced postoperative pain, and shorter procedure times [31]. The split-mouth clinical trial by Sadatmansouri et al. investigated the effects of CA adhesive on postoperative healing following periodontal surgery. Patients requiring surgical pocket elimination in at least two sextants were enrolled, with each mouth randomly assigned to a control group (sutures) or a test group (CA). At the one-week follow-up, no statistically significant differences were observed between the groups in terms of pain (control: 4.7 ± 1.34; test: 4.4 ± 1.68; *p* = 0.2), healing score (control: 3.3 ± 0.53; test: 2.7 ± 0.64; *p* = 0.3), or plaque index (control: 3.9 ± 0.82; test: 3.8 ± 0.97; *p* = 0.2). Similarly, probing depth at six weeks showed no significant difference (control: 2.5 ± 0.67; test: 2.8 ± 0.6; *p* = 0.2). Although no statistically significant differences were found, the findings suggest that CA adhesive may serve as a viable alternative to sutures, particularly in cases where patients cannot present for suture removal at the appointed time [32]. Khurana et al. compared the healing outcomes of isoamyl 2-CA and silk sutures following periodontal flap surgical procedures for pocket therapy in 20 patients. At 1 week, early healing was significantly better in the CA group (Group B) compared to the suture group (Group A) (*p* < 0.001), while no significant difference was observed at 2 weeks (*p* = 0.109). At 3 months, the plaque index improvement was 53.90% in Group A and 56.00% in Group B (*p* = 0.486), the bleeding index improvement was 99.20% and 100.00%, respectively (*p* = 0.324), and the probing depth reduction was 68.90% in Group A and 71.10% in Group B (*p* = 0.240), none of which was statistically significant. Pain and discomfort were significantly greater in the suture group (*p* = 0.001). All patients in the CA group reported esthetic satisfaction (*p* = 0.001), and no one experienced burning or itching in either group. Redness did not differ significantly between the groups (*p* = 0.147), and no cases showed crater formation or suture loss. Notably, all cases in Group A exhibited materia alba/debris, while none was observed in Group B (*p* = 0.001). These results suggest that isoamyl 2-CA promotes superior early healing, reduces postoperative discomfort, and improves esthetic and hygienic outcomes compared to silk sutures [33].

### 3.5. Socket Sealing in Alveolar Ridge Preservation

The study by Camacho-Alonso et al. included 140 patients who underwent alveolar ridge preservation using xenografts. In the control group, the socket was sealed with a collagen membrane (CMX), whereas, in the test group, CA (CX) was applied. Dental implants were placed immediately after extraction. After three months, the reduction in buccolingual alveolar ridge width was significantly greater in the CMX group compared to the CX group (*p* < 0.005), although no significant differences were found at later time points (*p* > 0.005). Clinical attachment level (CAL) values were significantly greater in the CMX group (*p* < 0.005), as was the marginal bone loss (MBL) (*p* < 0.001). Five cases of membrane exposure were recorded in the CMX group [34].

### 3.6. Wound Closure After Alveoloplasty

Narsingyani et al. compared N-butyl-2-CA and silk sutures for intraoral wound closure following bilateral alveoloplasty in 25 patients (50 sites). The CA group showed significantly better wound healing outcomes (*p* = 0.000) and faster closure time and hemostasis (*p* < 0.001 and *p* = 0.000, respectively). Postoperative edema was reduced in the CA group on days 1 and 3 (*p* < 0.05), and pain scores were also significantly reduced on days 3 (*p* = 0.000) and 7 (*p* = 0.009). Wound dehiscence occurred in 12% of suture sites and 4% of CA sites by day 7. Wound infection was observed in 8% of suture sites on day 3. Suture breakage was reported in three patients, while adhesive dislodgement occurred in two cases, one of which required reapplication. These results suggest that CA provides faster closure, reduced postoperative morbidity, and improved healing outcomes compared to conventional sutures [35]. In the study by Suthar et al., 20 patients requiring bilateral alveoloplasty in the same arch were included. Each patient received sutures on one side and CA adhesive on the other. Cyanoacrylate significantly reduced both the time required for wound closure (0.91 ± 0.37 vs. 3.77 ± 1.11 *min*; *p* < 0.001) and the time to achieve hemostasis (0.44 ± 0.23 vs. 2.71 ± 1.11 *min*; *p* < 0.001). A higher percentage of patients reported no postoperative pain on the CA side (58.8%) compared to the suture side (41.2%), although this difference was not statistically significant (*p* = 0.337). Wound healing scores were also slightly higher in the CA group, but the difference did not reach statistical significance (*p* = 0.289). These findings suggest that n-butyl-2-CA is an adequate alternative to conventional sutures in the closure of the surgical wound after alveoloplasty, and better than 3-0 silk sutures [36].

### 3.7. Soft Tissue Closure in Stage II Implant Recovery Procedure

The study by Kabilamurthi et al. evaluated the clinical effectiveness of isoamyl 2-CA compared to conventional black silk sutures for soft tissue closure in stage II implant recovery procedures. Twenty systemically healthy patients requiring second-stage implant surgery for a single posterior mandibular molar were enrolled in a single-blind randomized controlled trial. Ten patients received wound closure with black silk sutures (Group I), while the remaining ten were treated with isoamyl 2-CA (Group II). No statistically significant difference in healing was observed between the groups on day 3 (*p* = 0.10); however, by day 7, the CA group demonstrated significantly improved healing outcomes (*p* = 0.01). A significant difference in pain perception (VAS) was recorded on day 0 in favor of the CA group (*p* = 0.001), while no difference was found on day 3 (*p* = 0.31). These results suggest that isoamyl 2-cyanoacrylate may promote faster soft tissue healing and reduce immediate postoperative discomfort compared to silk sutures in implant stage II recovery [37].

### 3.8. JBI Critical Appraisal for Randomized Controlled Trials

All 19 included studies were appraised using the JBI Checklist for RCTs (https://jbi.global/sites/default/files/2020-08/Checklist_for_RCTs.pdf) (accessed on 8 July 2025). Most studies demonstrated overall good methodological quality, particularly in terms of the randomization procedures and baseline group comparability. However, participant and operator blinding was frequently absent, likely due to the surgical nature of the interventions. Outcome measures were generally applied consistently across studies. No studies were excluded based on the methodological quality (Table 2).

## 4. Discussion

This scoping review analyzed the clinical applications of CAs in oral surgery and periodontology, with particular attention given to their effects on postoperative outcomes such as pain, healing, and operative efficiency. One of the most recurrent findings in the literature is a significant reduction in operative time when CA is used instead of conventional sutures. Several studies highlighted this advantage, with Stavropoulou et al. and Karimi et al. reporting significantly shorter application times for CA compared to traditional suturing techniques [24,25,26].

Regarding pain management, numerous randomized clinical trials have shown that patients treated with CAs report less postoperative discomfort and require fewer analgesics than those treated with sutures. For instance, Alhourani et al. demonstrated that tissue adhesives led to shorter pain duration after free gingival grafting, while Tavelli et al. and Karimi et al. observed lower pain scores at palatal donor sites when CA was applied [19,20,21,22,23,24,25,26]. Furthermore, in the context of implant stage II recovery, Kabilamurthi et al. demonstrated that CA promoted faster soft tissue healing and reduced immediate postoperative pain when compared to conventional sutures [37].

Some studies have suggested that the effectiveness of CA in terms of postoperative outcomes may be enhanced when combined with other agents, such as PRF or hemostatic sponges. Both Tavelli et al. and Ozcan et al. reported that the association of CA with either PRF or collagen sponges contributed to reduced postoperative pain, bleeding, and patient discomfort, and improved healing [21,22,23].

However, not all studies found statistically significant differences when comparing CAs and sutures. In third molar surgeries, both Santmartí-Oliver et al. and Oladega et al. reported similar outcomes in terms of pain, swelling, trismus, and healing for the two closure methods [27,28]. Similarly, Gautam et al. [29] and Sadatmansouri et al. [32] observed comparable healing and pain levels in periodontal flap surgeries, although the CA groups required fewer analgesics [29]. By contrast, other studies highlighted the advantages of CA in the context of periodontal flap procedures, where it led to improved healing, reduced pain and discomfort, and enhanced esthetic outcomes compared to sutures. However, findings related to plaque accumulation were inconsistent [31,32,33].

Conversely, in the study by Leite et al., compared to other wound healing strategies, CA did not demonstrate superiority [7]. The same results were found in the meta-analysis by Li et al. [38], where both hyaluronic acid (HA) and PRF significantly enhanced wound healing by week 2 and reduced postoperative pain during week 1, with collagen sponges also improving pain scores, but CA showed no advantage in either healing outcomes or pain control [38].

In relation to periodontal flap surgery, the antimicrobial and hemostatic properties of CAs may offer additional clinical advantages. Gupta et al. found that CA-based dressings led to a more substantial reduction in Porphyromonas gingivalis levels and better pain scores than conventional dressings [30]. These findings are supported by the intrinsic bacteriostatic activity of CAs, especially 2-octyl derivatives, which inhibit Gram-positive and non-pseudomonas Gram-negative bacteria [39]. According to Romero et al., this antimicrobial effect may be attributed to the polymerization process of these adhesives [40]. Concerning hemostatic properties, Montanaro et al. showed that CA decreases the activated partial thromboplastin time (APTT), which promotes hemostasis and contributes to the adhesive properties [11]. These properties make CA particularly useful in surgical scenarios where bleeding control and quick closure are priorities.

Moreover, in grafting procedures, CA adhesives may offer advantages over conventional sutures, although the current findings are not consistent. While Alhourani et al. reported reduced graft shrinkage with tissue adhesive compared to sutures, Yılmaz et al. observed a greater horizontal graft dimension loss in the CA group [19,20,21,22,23,24,25].

Cyanoacrylates have been employed as sealants in alveolar ridge preservation, as demonstrated in a study by Camacho-Alonso et al. [34]. Compared to collagen membranes, they exhibited less buccolingual ridge resorption and marginal bone loss. These findings suggest that CA may offer better outcomes compared to collagen membranes in the context of alveolar ridge preservation.

In addition, CAs have shown promising results in alveoloplasty procedures. Narsingyani et al. [35] reported that, in patients undergoing bilateral alveoloplasty, CA achieved faster wound closure, superior healing, and reduced edema and pain compared to silk sutures, while Suthar et al. found that the tissue adhesive significantly shortened operative time and hemostasis duration, although the improvement in healing outcomes did not reach statistical significance [36].

In terms of toxicity, while there is no definitive evidence of carcinogenicity in humans, concerns have been raised about the cytotoxic effects of short-chain CAs. Longer-chain compounds, like n-butyl and 2-octyl CA, appear to be less toxic and more biocompatible due to slower degradation and reduced monomer release [14]. Cyanoacrylates can interact with biological tissues, releasing irritant fumes upon tissue contact due to the vaporization of monomers. These monomers rapidly polymerize in moist environments, reducing toxicity. Prolonged exposure may cause hypersensitivity (≈5% of cases), skin irritation, or, rarely, asthma [14]. Formaldehyde production during degradation has been proposed as a potential source of tissue irritation or hypersensitivity [10]. The in vitro findings reported by Mendoza et al. indicated decreased fibroblast activity following exposure to CA; however, no adverse effects on clinical wound healing were observed [41].

Some limitations of this study lie in the intrinsic nature of scoping reviews, which do not aim to quantitatively synthesize results or assess the quality of evidence, and in the heterogeneity among the selected studies in terms of surgical indications, CA formulations, and outcome measures, which limits direct comparison and generalizability. Furthermore, a number of trials had small sample sizes or lacked long-term follow-up. Future research should focus on conducting high-quality RCTs with larger sample sizes and standardized methodologies and outcome measures to assess the efficacy, safety, and long-term outcomes of CA adhesives across various dental surgical settings.

## 5. Conclusions

Overall, this review confirms that CA adhesives can be a valid alternative to other wound closure strategies for various dental surgical procedures. Their benefits are particularly evident in free gingival grafting and donor site management, where they reduce operative time, enhance patient comfort, and maintain favorable healing outcomes. Though they do not consistently show superiority in some procedures, such as third molar extractions or periodontal flap closures, the use of cyanoacrylates can still be considered a reliable option, especially when postoperative compliance or suture removal can be problematic. The current evidence remains limited by small sample sizes, the heterogeneity of CA formulations, and short follow-up periods.

## Figures and Tables

**Table 1:** Summary of studies included in the review after performing the search strategy for studies exploring CAs in oral surgery and periodontology.

Authors (Year)	Title	Application Field	Summary of Results
Alhourani et al. (2022) [19]	Comparative study between using a tissue adhesive (N-BCA & OCA) and surgical sutures in free gingival graft surgery: A randomized controlled clinical trial	Free gingival graft surgery	The CA group exhibited significantly less graft shrinkage, improved healing scores, and reduced postoperative pain compared to the suture group.
Basma et al. (2023) [20]	Patient-reported outcomes of palatal donor site healing using four different wound dressing modalities following free epithelialized mucosal grafts: A four-arm randomized controlled clinical trial.	Palatal wound healing	No differences among the test groups. All tested dressing modalities, including the collagen plug combined with CA, effectively reduced donor site pain compared to the pain of the control group (collagen plug combined with sutures). The palatal stent achieved the lowest pain scores.
Tavelli et al. (2018) [21]	Minimizing Patient Morbidity Following Palatal Gingival Harvesting: A Randomized Controlled Clinical Study	Palatal wound healing	The combination of a gelatin sponge and CA significantly minimized postoperative pain and improved healing compared to sutures alone.
Tavelli et al. (2019) [22]	Pain perception following epithelialized gingival graft harvesting: a randomized clinical trial	Palatal wound healing	Application of CA over a collagen sponge significantly decreased pain perception and analgesic intake following gingival grafting.
Ozcan et al. (2017) [23]	Effects of Platelet-Rich Fibrin on Palatal Wound Healing After Free Gingival Graft Harvesting: A Comparative Randomized Controlled Clinical Trial	Palatal wound healing	The PRF + CA group demonstrated statistically significant improvements for all the parameters assessed (bleeding, pain, epithelialization, feeding habits, and sensation scores) compared to the groups treated with cyanoacrylate or gauze alone.
Stavropoulou et al. (2019) [24]	A randomized clinical trial of cyanoacrylate tissue adhesives in donor site of connective tissue grafts	Palatal wound healing	No significant difference in patient-reported discomfort, patient self-reported pain, analgesic consumption, or MEHI between CA and PTFE sutures. There was reduced application time in the CA group.
Yılmaz et al. (2022) [25]	The Effects of Cyanoacrylate on Clinical Healing and Self-Reported Outcomes Following Free Gingival Graft Surgery: A Randomized Clinical Study	FGG stabilization and palatal wound healing	CA was safe and comparable to sutures, although greater horizontal graft dimension loss was observed in the cyanoacrylate group.
Karimi et al. (2024) [26]	Effect of Cyanoacrylate Adhesive on Palatal Wound Healing Following Free Gingival Grafting: A Clinical Trial	Palatal wound healing	CA-treated sites exhibited significantly lower pain and better healing, requiring reduced analgesic use and a shorter application time than sutures.
Santmartí-Oliver et al. (2024) [27]	Cyanoacrylate versus suture as flap closure methods in mandibular third molar surgery: a split-mouth randomized controlled clinical study	Flap closure in third molar surgery	No significant differences in postoperative outcomes (pain, swelling, trismus, and healing) or patient-reported outcome measures (PROMs) between CA and silk sutures in third molar surgery.
Oladega et al. (2019) [28]	Cyanoacrylate tissue adhesive or silk suture for closure of surgical wound following removal of an impacted mandibular third molar: A randomized controlled study	Flap closure in third molar surgery	CA-treated wounds showed significantly less bleeding on the first postoperative day, with similar outcomes in terms of pain, swelling, trismus, wound dehiscence, and infection.
Gautam et al. (2024) [29]	Beyond the Needle: A Comparative Evaluation of Silk Sutures and Cyanoacrylate for Periodontal Flap Closure	Periodontal flap closure	The CA group and suture group exhibited comparable healing and pain levels; however, the cyanoacrylate group required fewer postoperative analgesics.
Gupta et al. (2025) [30]	Assessment of Efficacy of Two Different Periodontal Dressing Materials Following Open Flap Debridement: A Clinical Study	Periodontal flap closure	The CA dressing showed superior antimicrobial activity and prompted less pain compared to Coe-Pak™ and sutures.
Chandra et al. (2021) [31]	Comparative Evaluation of N-Butyl Cyanoacrylate with Braided Silk Suture after Periodontal Flap Surgery: A Randomized Controlled Trial	Periodontal flap closure	CA improved early healing, reduced plaque accumulation and inflammation, and minimized pain in the early postoperative period.
Sadatmansouri et al. (2020) [32]	Effect of Cyanoacrylate Adhesive on Tissue Healing After Periodontal Surgery	Periodontal flap closure	No statistically significant differences in pain, healing, plaque index, or probing depth between CA and sutures.
Khurana et al. (2016) [33]	Comparative evaluation of healing after periodontal flap surgery using isoamyl 2-cyanoacrylate (bioadhesive material) and silk sutures: A split-mouth clinical study	Periodontal flap closure	CA resulted in significantly better early healing, reduced pain and discomfort, and improved esthetic outcomes. No statistically significant differences were observed in plaque index, bleeding index, or probing depth.
Camacho-Alonso et al. (2024) [34]	Cyanoacrylate versus Collagen Membrane as a Sealing for Alveolar Ridge Preservation: A Randomized Clinical Trial	Sealing for alveolar ridge preservation	Collagen membrane resulted in greater buccolingual ridge resorption and marginal bone loss than CA.
Narsingyani et al. (2023) [35]	Attached Oral Mucosal Wound Closure using Blue Glue—A Prospective Clinical Study	Wound closure after alveoloplasty	CA achieved faster wound closure, superior healing, and reduced edema and pain compared to silk sutures.
Suthar et al. (2020) [36]	Comparing intra-oral wound healing after alveoloplasty using silk sutures and n-butyl-2-cyanoacrylate	Wound closure after alveoloplasty	CA significantly reduced operative time and hemostasis duration. Healing outcomes were slightly improved but not statistically significant.
Kabilamurthi et al. (2022) [37]	Comparison of effectiveness of cyanoacrylate over conventional sutures in Implant stage II recovery procedure	Implant stage II recovery	CA provided faster soft tissue healing and reduced early postoperative pain compared to conventional sutures in implant stage II recovery.

**Table 2:** JBI critical appraisal of the included RCT studies. Each study was assessed across 13 criteria, indicating one of the following responses: Yes, No, Unclear, and Not Applicable (NA).

Authors (Year)	Q1	Q2	Q3	Q4	Q5	Q6	Q7	Q8	Q9	Q10	Q11	Q12	Q13	Overall Appraisal
*Alhourani* et al. *(2022)* [19]	Yes	Yes	Yes	No	No	Yes	Yes	Yes	Yes	Yes	Yes	Yes	Yes	Include
*Basma* et al. *(2023)* [20]	Yes	Yes	Yes	No	No	No	Yes	Yes	Yes	Yes	Yes	Yes	Yes	Include
*Tavelli* et al. *(2018)* [21]	Yes	Yes	Yes	No	No	Yes	Yes	Yes	Yes	Yes	Yes	Yes	Yes	Include
*Tavelli* et al. *(2019)* [22]	Yes	Yes	Yes	Yes	Yes	yes	Yes	Yes	Yes	Yes	Yes	Yes	Yes	Include
*Ozcan* et al. *(2017)* [23]	Yes	Yes	Yes	No	Yes	yes	Yes	Yes	Yes	Yes	Yes	Yes	Yes	Include
*Stavropoulou* et al. *(2019)* [24]	Yes	Yes	Yes	No	No	Yes	Yes	Yes	Yes	Yes	Yes	Yes	Yes	Include
*Yılmaz* et al. *(2022)* [25]	Yes	Yes	Yes	No	No	Yes	Yes	Yes	Yes	Yes	Yes	Yes	Yes	Include
*Karimi* et al. *(2024)* [26]	Yes	Yes	Yes	No	No	Yes	Yes	Yes	Yes	Yes	Yes	Yes	Yes	Include
*Santmartí-Oliver* et al. *(2024)* [27]	Yes	Yes	Yes	No	No	Yes	Yes	Yes	Yes	Yes	Yes	Yes	Yes	Include
*Oladega* et al. *(2019)* [28]	Yes	Yes	Yes	Yes	No	Yes	Yes	Yes	Yes	Yes	Yes	Yes	Yes	Include
*Gautam* et al. *(2024)* [29]	Yes	Yes	Yes	No	No	No	Yes	Yes	Yes	Yes	Yes	Yes	Yes	Include
*Gupta* et al. *(2025)* [30]	Yes	Yes	Yes	No	No	No	Yes	Yes	Yes	Yes	Yes	Yes	Yes	Include
*Chandra* et al. *(2021)* [31]	Yes	Yes	Yes	No	No	No	Yes	Yes	Yes	Yes	Yes	Yes	Yes	Include
*Sadatmansouri* et al. *(2020)* [32]	Yes	Yes	Yes	No	No	No	Yes	Yes	Yes	Yes	Yes	Yes	Yes	Include
*Khurana* et al. *(2016)* [33]	Yes	Yes	Yes	No	No	No	Yes	Yes	Yes	Yes	Yes	Yes	Yes	Include
*Camacho-Alonso* et al. *(2024)* [34]	Yes	Yes	Yes	No	No	Yes	Yes	Yes	Yes	Yes	Yes	Yes	Yes	Include
*Narsingyani* et al. *(2023)* [35]	Yes	Yes	Yes	No	No	Yes	Yes	Yes	Yes	Yes	Yes	Yes	Yes	Include
*Suthar* et al. *(2020)* [36]	Yes	Yes	Yes	No	No	Yes	Yes	Yes	Yes	Yes	Yes	Yes	Yes	Include
*Kabilamurthi* et al. *(2022)* [37]	Yes	Yes	Yes	No	No	Yes	Yes	Yes	Yes	Yes	Yes	Yes	Yes	Include

## Data Availability

No new data were created or analyzed in this study.
